# Leucine aminopeptidase may contribute to the intrinsic resistance of cancer cells toward cisplatin as revealed by an ultrasensitive fluorescent probe†
†Electronic supplementary information (ESI) available: Experimental section and supporting figures. See DOI: 10.1039/c5sc03600c



**DOI:** 10.1039/c5sc03600c

**Published:** 2015-10-22

**Authors:** Qiuyu Gong, Wen Shi, Lihong Li, Huimin Ma

**Affiliations:** a Beijing National Laboratory for Molecular Sciences , Key Laboratory of Analytical Chemistry for Living Biosystems , Institute of Chemistry , Chinese Academy of Sciences , Beijing 100190 , China . Email: mahm@iccas.ac.cn

## Abstract

Cisplatin, a typical anticancer drug, is often used to treat different cancers, and leucine aminopeptidase (LAP) is known to be widely distributed in organisms from bacteria to humans, including various cancer cells. However, cancer cells display different intrinsic or acquired resistance toward cisplatin, and it is unclear whether intracellular LAP plays a role in the intrinsic drug resistance, mainly due to the lack of a sensitive detection approach for LAP because this enzyme usually exists at trace levels in cancer cells. Herein, by developing an ultrasensitive LAP fluorescent probe (detection limit 0.42 ng mL^–1^) and combining it with confocal fluorescence imaging, we analyze the concentration change of LAP in cancer cells such as HepG2 and A549 cells under cisplatin treatment. We find that a large increase in the LAP concentration occurs in HepG2 rather than in A549 cells. These different changes are further confirmed by an ELISA kit. A cell viability assay reveals that HepG2 cells with a higher level of LAP have much stronger resistance toward cisplatin than A549 cells, suggesting that LAP may serve as a simple indicator to reflect the relative resistance of different cancer cells. Importantly, inhibiting the expression of LAP with siRNA further decreases cell viability. These findings support that LAP may contribute to the intrinsic resistance of cancer cells toward cisplatin. In addition, the proposed probe may find more uses in studying the cellular LAP function, and improving chemotherapeutic cancer treatment.

## Abstract 2

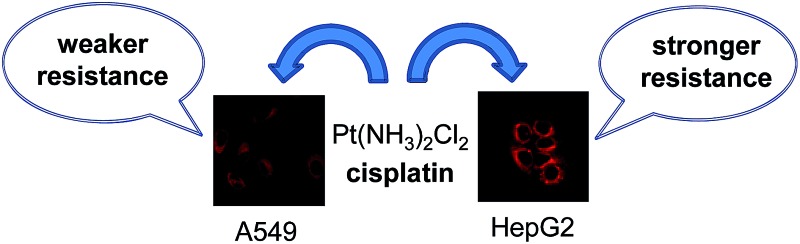
Leucine aminopeptidase may contribute to the intrinsic resistance of cancer cells toward cisplatin as revealed by an ultrasensitive fluorescent probe.

## Introduction

Cisplatin, a typical anticancer drug, has been extensively used in cancer chemotherapy.^1^ However, cancer cells display different intrinsic or acquired resistance to this anticancer drug,^2^ and whether some enzymes contribute to the intrinsic resistance of cancer cells toward cisplatin is poorly understood. Leucine aminopeptidase (LAP), a key enzyme capable of affecting diverse biological and physiological processes,^3–6^ is known to widely exist in organisms from bacteria to humans, including various cancer cells.^7,8^ Moreover, LAP may be a kind of resistance factor in cancer cells.^7,9^ Unfortunately, the relationship between LAP and the intrinsic drug resistance of cancer cells is unknown. Therefore, our interest is to explore whether LAP plays a role in the intrinsic drug resistance of cancer cells toward cisplatin. To accomplish this purpose, the major challenge we confront is how to achieve the sensitive detection of LAP and in particular how to monitor its subtle concentration change under stimulation from cisplatin because this intracellular enzyme usually exists at trace levels in cancer cells.^10,11^ Herein, by developing a new ultrasensitive fluorescent LAP probe and combining it with confocal fluorescence imaging, the changes of the LAP concentrations in cancer cells such as HepG2 and A549 cells before and after treatment with cisplatin are determined, which shows that a large increase in the LAP concentration occurs in HepG2 rather than in A549 cells. This observation is also confirmed by an ELISA kit. Further studies on the relationship between the intracellular LAP concentrations and the cell viabilities reveal that LAP indeed plays a role in the intrinsic resistance of cancer cells toward cisplatin. Herewith we report these results.

For LAP assays in living cells, fluorescent probes have attracted much more attention because of their excellent temporal-spatial resolution, high sensitivity and good selectivity.^5,8,12–18^ The general design strategy for a fluorescent probe is to incorporate a recognition moiety into a fluorescent skeleton, and an ideal fluorescent probe for a given analyte should have excellent analytical performance with a simple synthetic route. However, with existing LAP probes it is still difficult to monitor the subtle change of the intracellular LAP concentration due to their insufficient sensitivity (detection limit > 1 ng mL^–1^), and some of the probes cannot be used in living cells because of their short excitation and emission wavelengths. Therefore, a new fluorescent LAP probe with both large analytical wavelengths and super-high sensitivity is urgently needed.

**Fig. 1:**
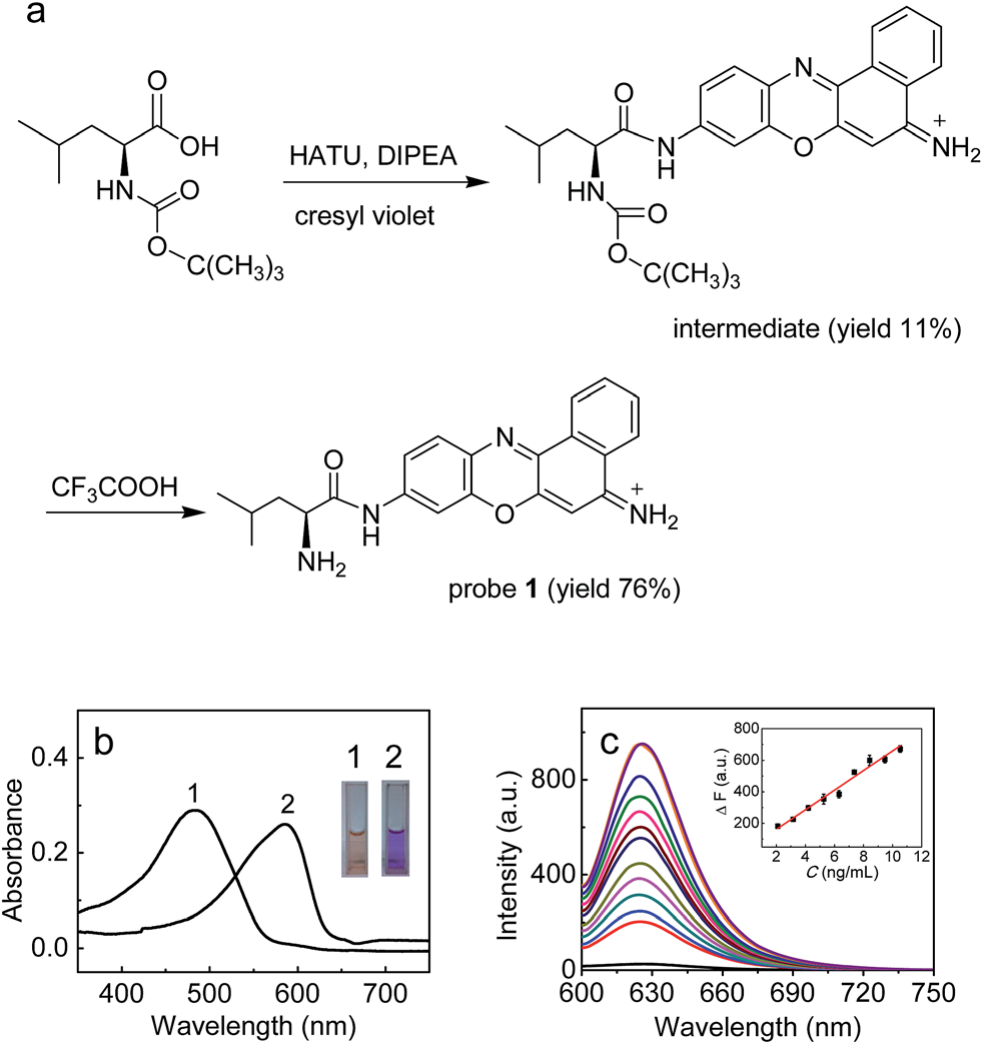
(a) Synthesis of probe **1**. (b) Absorption spectra of **1** (10 μM) before (curve 1) and after (curve 2) reaction with LAP (26 ng mL^–1^). The inset shows the corresponding color change. (c) Fluorescence response of **1** (10 μM) to LAP at different concentrations (from bottom to top: 0–26 ng mL^–1^). The inset shows the plot of Δ*F versus* the LAP concentration. *λ*_ex/em_ = 585/625 nm.

In this work, with these criteria in mind, we have developed such a probe (**1**; Fig. 1) by incorporating l-leucine as a recognition moiety into the skeleton of cresyl violet. The design of the probe was based on the following three considerations. First, long analytical wavelengths: cresyl violet was chosen as the fluorochrome because of its long wavelengths (*λ*_ex_/*λ*_em_ = 585/625 nm) as well as high fluorescence quantum yield and excellent cell membrane permeability;^19^ second, high sensitivity: the substitution of the amino group of cresyl violet can efficiently quench its fluorescence, which could make the resulting probe possess a low background signal, thereby benefiting a fluorescence off–on response and achieving high detection sensitivity;^20^ third, simplicity and practicability: l-leucine as a specific LAP substrate^8,13^ can be directly coupled to the amino group of cresyl violet through an amide bond, which makes the preparation of the probe rather facile. As shown in Fig. 1a, probe **1** can be readily prepared by treating cresyl violet with the protected l-leucine, and then removing the protecting group in the presence of trifluoroacetic acid (ESI†). The obtained probe was well characterized by NMR and mass spectral analyses (Fig. S1–S4†).

## Results and discussion

### Spectroscopic response of probe **1** to LAP

The spectroscopic properties and analytical performance of probe **1** were studied (Fig. 1; see also ESI† for details). As is seen from Fig. 1b, probe **1** exhibits a strong absorption peak at about 494 nm, but its reaction with LAP produces a red-shifted absorption peak at about 585 nm, with a significant color change from orange to purple (the inset of Fig. 1b). Notably, probe **1** has a rather low background signal (*Φ* ≈ 0.02), but its reaction with LAP leads to a large fluorescence enhancement. As expected, this distinct off–on response favors sensitive detection. It is worth noting that the absorption and fluorescence spectra from the reaction system accord well with those from cresyl violet.^20*c*^ This suggests that the enzymatic cleavage reaction of **1** by LAP occurred and caused the release of cresyl violet, which was verified by mass spectral analysis (*m*/*z* = 262.1 [M]^+^; Fig. S5†). The introduction of ubenimex, an inhibitor of LAP,^21^ into the reaction system markedly decreases the fluorescence (Fig. S6a in ESI;† it should be noted that ubenimex at a concentration of <1 μM had no obvious inhibition effect); moreover, ubenimex hardly affects the fluorescence intensity of cresyl violet (Fig. S6b†). These results further support that the fluorescence off–on response is attributed to the action of LAP. Under the optimized conditions (reaction at 37 °C for 20 min in 6.7 mM PBS of pH 7.4; Fig. S7 and S8 in ESI†), probe **1** exhibits a good linear fluorescence response to LAP in the concentration range of 2–12 ng mL^–1^ (inset of Fig. 1c), with the linear equation of Δ*F* = 62.21 × *C* (ng mL^–1^) + 36.6 (*R* = 0.993), where Δ*F* is the difference of the fluorescence intensity of **1** after and before reacting with LAP. Following the previous method,^22^ the detection limit is determined to be 0.42 ng mL^–1^ for LAP, which is the lowest one reported so far to our knowledge (Table S1†). This may enable the probe to detect trace amounts of intracellular LAP. According to the Michaelis–Menten equation (Fig. S9†),^23^ the corresponding Michaelis constant (*K*_m_) and the maximum of the initial reaction rate (*V*_max_) for the present enzymatic reaction were determined to be 46.7 μM and 0.20 μM s^–1^. Furthermore, probe **1** displays high selectivity for LAP over various other substances (including biothiols and co-existing enzymes) at their physiological concentrations (Fig. S10†). These findings indicate that probe **1** is an excellent specific substrate for LAP.

### Detection of LAP in human liver microsomes

**Fig. 2:**
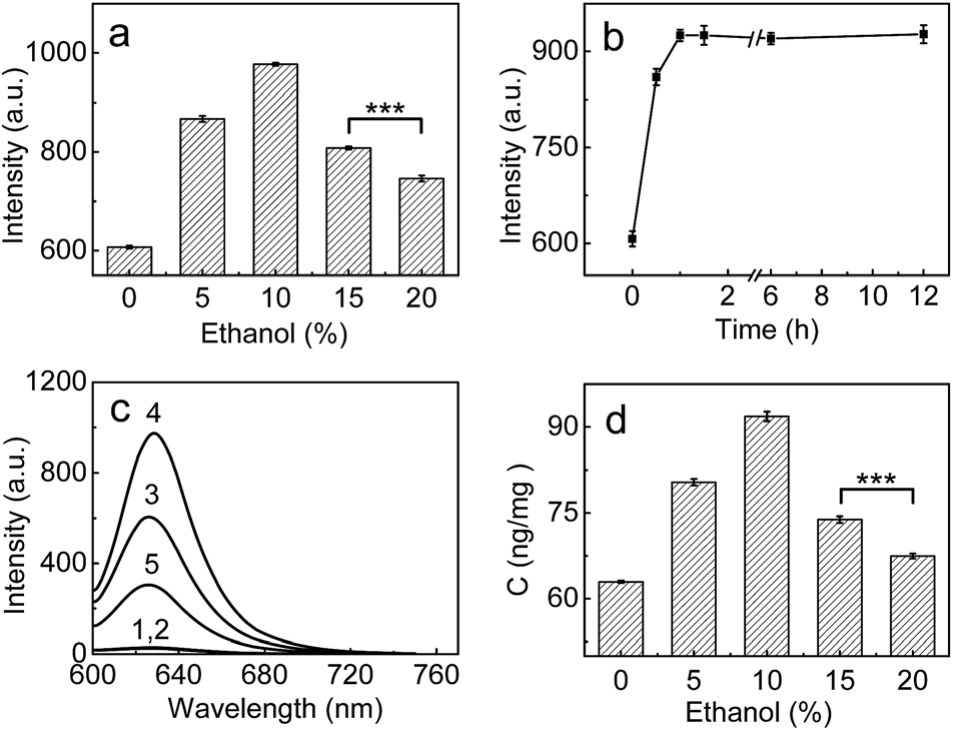
(a) Changes of the fluorescence intensity from the reaction system of **1** (10 μM) and microsomes (0.25 mg mL^–1^) in the presence of different concentrations of ethanol. (b) Effect of time on the fluorescence intensity of the reaction system (10 μM **1** + 0.25 mg mL^–1^ microsomes) in the presence of 10% ethanol. (c) The fluorescence intensity of different systems: (1) 10 μM probe; (2) 10 μM probe + 10% ethanol; (3) 10 μM probe + 0.25 mg mL^–1^ microsomes; (4) system 3 + 10% ethanol; (5) system 4 + 1 mM ubenimex. (d) The change of the LAP concentration in the microsomes treated with various ethanol concentrations (0–20%); the LAP concentrations in the microsomes were determined by an ELISA kit (see ESI†). *λ*_ex/em_ = 585/625 nm; ***p < 0.001, two-sided Student's *t*-test.

Having demonstrated the superior analytical performance of **1** (in particular its super-high sensitivity), the probe was then preliminarily investigated for its ability to monitor the change in trace amounts of LAP in a known model;^24^ namely, whether ethanol could increase LAP activity/concentration. As can be seen from Fig. 2a, the fluorescence intensity of the microsomes treated with ethanol is larger than that of the microsomes without ethanol (control), and the maximum difference (about a 61% increase in fluorescence intensity) is observed with an ethanol concentration of 10%. This fluorescence increase reflects the increase in the LAP activity/concentration, which is in agreement with previous findings.^24^ Interestingly, the fluorescence intensity decreases gradually with the increase of ethanol concentration over 10%. The reason for this behavior is unclear, but a possible explanation may be due to the fact that higher concentrations (*e.g.*, 20%) of ethanol may lead to the denaturation of LAP, as evidenced by the significant decrease in the intensity of the negative peak at about 220 nm and thus the destruction of the typical α-helix structure of LAP in the circular dichroism (CD) spectral analyses (Fig. S11 in ESI†). The effect of the exposure time was studied by treating the microsomes with 10% ethanol for different periods of time, which indicates that the maximum activity of LAP can be achieved after 60 min (Fig. 2b). On the other hand, inhibitor experiments (ubenimex) were performed. As shown in Fig. 2c, the introduction of ubenimex leads to much weaker fluorescence. Furthermore, ethanol itself hardly affects the fluorescence of both probe **1** and cresyl violet (Fig. S12 in ESI†), and the increase in LAP concentration in the ethanol-treated microsomes was further confirmed by an ELISA kit (Fig. 2d). The above results indicate that the fluorescence enhancements are indeed caused by LAP, and changes in the LAP concentration at trace levels can be detected by our ultrasensitive probe.

### Detection of LAP in living cells

Next, the capability of **1** for detecting endogenous LAP in cancer cells such as HepG2 and A549 cells was examined *via* confocal fluorescence imaging. As shown in Fig. S13 and S14 (ESI†), HepG2 and A549 cells themselves show almost no background fluorescence. However, the cells incubated with **1** show strong fluorescence (Fig. S13b and S14b†), suggesting that the probe has good cell-permeability and is capable of detecting endogenous intracellular LAP. It is noted that the relative pixel intensity from the HepG2 cells is about two times higher than that from the A549 cells under the same fluorescence imaging conditions (Fig. S15†), suggesting that HepG2 cells have a higher level of intracellular LAP. Furthermore, the presence of trace amounts of LAP in these cells was verified with an ELISA kit, and the obtained results also supported that HepG2 cells have about 2-fold more LAP than A549 cells (*vide infra*). On the other hand, the cells pretreated with the ubenimex inhibitor showed a markedly decreased fluorescence (Fig. S13c and S14c, and Fig. S13g and S14g†), further indicating that the fluorescence change of **1** in the cells arises indeed from LAP. In addition, probe **1** displays good biocompatibility (Fig. S16 in ESI†).

**Fig. 3:**
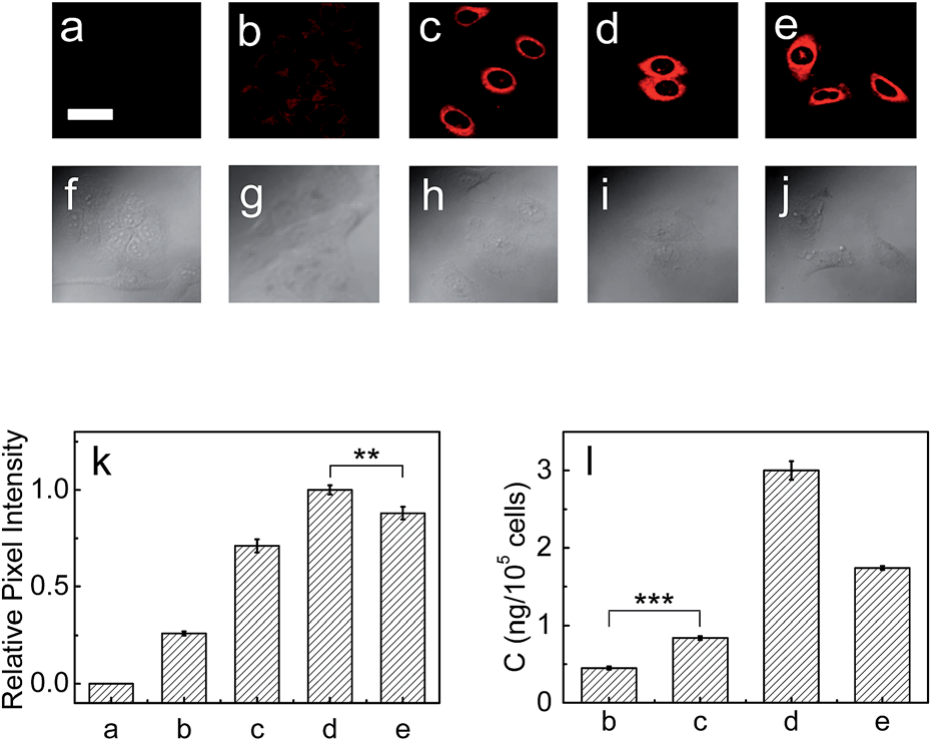
Fluorescence images of LAP in HepG2 cells. (a) HepG2 cells only. The HepG2 cells were pretreated with cisplatin at various concentrations (0, 0.5, 1.0, 2.0 mg L^–1^ for images (b)–(e), respectively) for 12 h, and then incubated with the probe (10 μM) for 20 min. Scale bar 20 μm. (f)–(j) Differential interference contrast (DIC) images of the above corresponding HepG2 cells. (k) Relative pixel intensity measurements (*n* = 3) from images (a)–(e) by using the software ImageJ (the pixel intensity from image (d) is defined as 1.0). (l) The concentrations of LAP in the above HepG2 cells determined by an ELISA kit. ***p < 0.001, **p < 0.01, two-sided Student's *t*-test.

Then, the effects of cisplatin on the concentration change of intracellular LAP in HepG2 and A549 cells were studied by using probe **1**. As can be seen from Fig. 3, the fluorescence intensity of the HepG2 cells increases with the increase of cisplatin in the concentration range of 0–1 mg L^–1^ (images (b)–(d) and Fig. 3k), which suggests the gradual increase of the intracellular LAP concentration. However, higher concentrations (*e.g.*, 2.0 mg L^–1^) of cisplatin decrease the fluorescence intensity (as can be seen by comparing images (d) and (e); Fig. 3k), implying a decrease of the LAP concentration. Though the reason for this phenomenon is unclear and needs to be investigated in the future, we speculate that it may be related to the intrinsic drug resistance of cancer cells. The changes of the LAP concentration in the HepG2 cells were also determined by an ELISA kit (Fig. 3l), which clearly supports our above results. Similarly, treatment of the A549 cells with cisplatin also caused an increase in the intracellular LAP concentration, but such an increase is not significant at the 99% confidence level using the two-sided Student's *t*-test, when compared with the control group (Fig. S17 in ESI†). The effects of the cisplatin incubation time on the concentration change of LAP in HepG2 cells were then studied. As shown in Fig. S18,† with the increase of the cisplatin incubation time, the fluorescence intensity of the HepG2 cells gradually increases, and after 6 h the fluorescence intensity, representing the intracellular LAP activity/concentration, reaches a maximum (images (b)–(f) and Fig. S18o in ESI†). This increase in the intracellular LAP activity is further supported by the inhibitor experiment with ubenimex, that is, the introduction of ubenimex into the HepG2 cells leads to much weaker fluorescence (Fig. S18g†). Importantly, cisplatin scarcely affects the fluorescence of **1** and cresyl violet as well as the activity and structure of LAP (Fig. S19†); moreover, cisplatin does not alter the background fluorescence of the HepG2 cells (Fig. S20†). These results indicate that cisplatin induces the up-regulation of intracellular LAP, and the probe can be used to monitor this increase of LAP.

### The relationship between LAP and intrinsic drug resistance

**Fig. 4:**
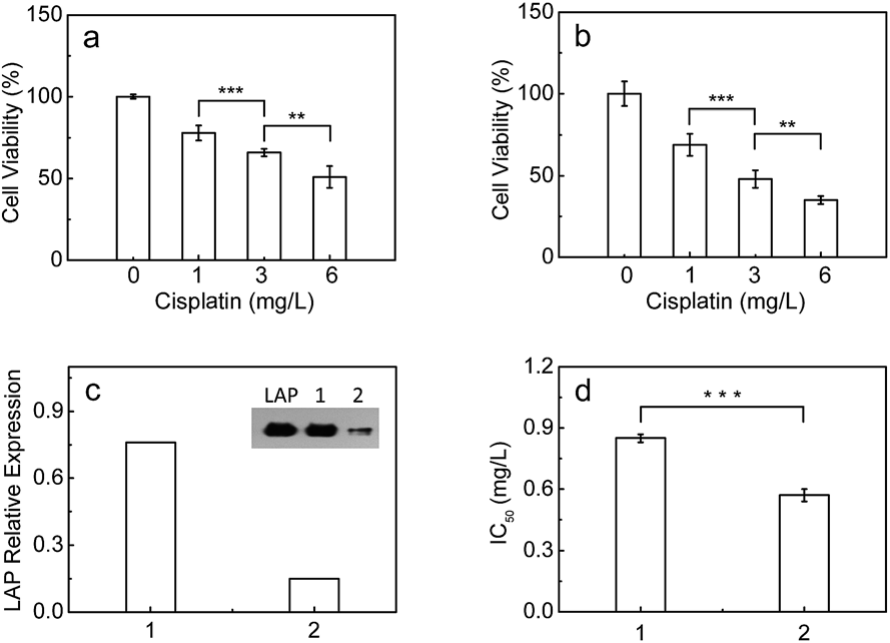
The cell viability of (a) HepG2 and (b) A549 cells with various concentrations of cisplatin (0–6 mg mL^–1^). (c) The LAP relative expressions in (1) HepG2 and (2) siRNA-transfected HepG2 cells. The inset shows the corresponding protein levels as well as the LAP standard by western blot analyses. (d) IC_50_ values of cisplatin for (1) HepG2 and (2) siRNA-transfected HepG2 cells. The cells were treated with cisplatin for 72 h. ***p < 0.001, **p < 0.01, two-sided Student's *t*-test.

It has been found that LAP c-DNA transfected endometrial adenocarcinoma cells show strong resistance toward cisplatin.^7^ As mentioned above, however, it is unclear whether the endogenous intracellular LAP plays a role in the intrinsic resistance of cancer cells toward cisplatin. Therefore, the standard MTT [3-(4,5-dimethylthiazol-2-yl)-2,5-diphenyltetrazolium bromide] assay was used to investigate the effect of the intracellular LAP concentrations on the cell viabilities (ESI†). As shown in Fig. 4a and b, the cell viability of both the HepG2 and A549 cells decreases gradually with the increase of cisplatin from 1 to 6 mg L^–1^, but the HepG2 cells with a higher level of endogenous LAP (0.46 ng per 10^5^ cells, determined by ELISA kit; Fig. 3l) have a much stronger resistance than the A549 cells (0.22 ng LAP per 10^5^ cells; Fig. S17l†). For instance, the cell viability of the HepG2 cells is about 66% at 3 mg L^–1^ cisplatin, which is much larger than that (48%) of the A549 cells. Importantly, we inhibited the expression of LAP in the HepG2 cells using siRNA (Fig. 4c), achieving a significantly decreased IC_50_ value of cisplatin for cell growth (Fig. 4d). This implies that cancer cells with higher levels of intracellular LAP may have stronger intrinsic resistance. In other words, LAP may contribute to the intrinsic resistance of cancer cells toward cisplatin, possibly through altering the intracellular interactive pathways (*e.g.*, transfer and distribution) of the drug.

## Conclusions

In conclusion, we have developed a new ultrasensitive LAP fluorescent probe, which can be used to monitor the concentration changes of trace amounts of LAP in different biosamples such as human liver microsomes and cancer cells. Moreover, we find that cancer cells with a higher level of LAP display much stronger resistance toward cisplatin, implying that LAP may contribute to the intrinsic resistance and may serve as a simple indicator to reflect the relative resistance of different cancer cells. This study provides a useful reference for exploring the action of other enzymes on the intrinsic resistance of cancer cells. In addition, it is believed that the proposed probe may find more uses in studying the cellular LAP function, and improving chemotherapeutic cancer treatment.
