# Supplementary material for: Leucine aminopeptidase may contribute to the intrinsic resistance of cancer cells toward cisplatin as revealed by an ultrasensitive fluorescent probe
Source: Chem Sci. 2015 Oct 22;7(1):788–92. doi: 10.1039/c5sc03600c (PMC5580032; doi:10.1039/c5sc03600c)
Supplement: Supplementary file 1 [file SC-007-C5SC03600C-s001.pdf]

Electronic Supplementary Material (ESI) for Chemical Science.
This journal is © The Royal Society of Chemistry 2015

Electronic Supplementary Information for:

Leucine Aminopeptidase May Contribute to Intrinsic Resistance of Cancer Cells toward Cisplatin as Revealed by an Ultrasensitive Fluorescent Probe

Qiuyu Gong, Wen Shi, Lihong Li and Huimin Ma*

Beijing National Laboratory for Molecular Sciences, Key Laboratory of Analytical Chemistry for Living Biosystems, Institute of Chemistry, Chinese Academy of Sciences, Beijing 100190, China. E-mail: mahm@iccas.ac.cn

1. Experimental

Apparatus and reagents. Fluorescence measurements were made on a Hitachi F-2500 fluorimeter (Tokyo, Japan). A model HI-98128 pH-meter (Hanna Instruments Inc., USA) was used for pH measurements. ^{1}H NMR and ^{13}C NMR spectra were measured with a Bruker Avance 400 spectrometer in CD_3OD. Electrospray ionization mass spectra (ESI-MS) were measured with an LC-MS 2010A (Shimadzu) instrument. High resolution electrospray ionization mass spectra (HR-ESI-MS) were recorded on an APEX IV FTMS instrument (Bruker, Daltonics). Absorption spectra were measured in 1-cm quartz cells with a TU-1900 spectrophotometer (Beijing, China). Cell imaging experiments were operated on a FV 1000-IX81 confocal laser scanning microscope (Olympus, Japan). Enzyme linked immunosorbent assay (ELISA) was operated on a Microtiter plate assay system (Molecular Devices, USA). Circular dischroism (CD) spectra were recorded on a J-815 CD spectrometer (JASCO, Japan). The fluorescence quantum yield (Φ) was determined by using fluorescein (Φ = 0.85 in 0.1 M NaOH) as a standard (Zhang *et al. J. Am. Chem. Soc.* **2011**, *133*, 14109). The Western blot signal was detected using an enhanced chemiluminescence (ECL) kit from Bio-Rad, USA.

Cresyl violet acetate, N,N-diisopropylethylamine (DIPEA), O-(7-aza-1H-benzotriazol-1-yl)-N,N,N',N'-tetramethyluronium hexafluorophosphate (HATU) and N-t-BOC-L-leucine monohydrate were purchased from Acros Organics. Trifluoroacetic acid (HPLC grade) was purchased from Alfa Aesar Chemicals. Reduced glutathione (GSH), cisplatin [*cis*-dichlorodiamineplatinum(II)], leucine aminopeptidase (LAP), esterase, dimethyl sulfoxide (DMSO), glutamic acid dehydrogenase and reduced nicotinamide adenine

dinucleotide (NADH) were purchased from Sigma-Aldrich. Dulbecco's Modified Eagle's medium (DMEM), HepG2 and A549 cell lines were purchased from KeyGEN BioTECH Co. LTD, Nanjing, China. A phosphate buffered saline (PBS: 137 mM NaCl, 2.7 mM KCl, 4.3 mM Na_2HPO_4 and 1.4 mM KH_2PO_4) of pH 7.4 was obtained from MatTek Co. Fetal bovine serum was purchased from Invitrogen Corporation. 3-(4,5-Dimethylthiazol-2-yl)-2,5-diphenyltetrazolium bromide (MTT) was obtained from Serva Electrophoresis GmbH. LAP BioAssay™ ELISA kit (Human) was purchased from Lengton Bioscience Co. LTD, Shanghai, China. Radio-Immunoprecipitation Assay (RIPA) Lysis Buffer was purchased from CWbiotech. Co. LTD, Beijing, China. Proteins were pure as judged by Coomassie-stained SDS-polyacrylamide gel electrophoresis (SDS-PAGE). Fetal bovine serum (FBS) was obtained from ExCell Biology, USA. Anti-LAP antibody was purchased from proteintech™, USA. All other chemicals used were local products of analytical grade. Ultrapure water (over 18 MΩ·cm) from a Milli-Q reference system (Millipore) was used throughout. The stock solution (1.0 mM) of probe **1** was prepared by dissolving requisite amount of it in DMSO. Stock solutions of other substances were prepared by dissolving in PBS or water.

Synthesis of probe 1. N-t-BOC-L-Leucine monohydrate (249 mg, 1.0 mmol), HATU (570 mg, 1.5 mmol) and DIPEA (2.0 mmol, 435 μL) were added to 30 mL of CH_2Cl_2 at 0 °C. After stirring for 40 min, a suspension of cresyl violet acetate (321 mg 1.0 mmol) in 10 mL of CH_2Cl_2 was added slowly, and the reaction mixture was further stirred at room temperature for 3 h. Then, the mixture was diluted with CH_2Cl_2 (50 mL), and washed three times with water (100 mL×3). The solvent was removed by evaporation under reduced pressure, and the crude product was purified by silica gel chromatography eluted with CH_2Cl_2/methanol (v/v, 10/1), affording the intermediate as a red solid (50 mg, yield 10.5%). The ^{1}H NMR and ^{13}C NMR spectra of the intermediate are shown below in Figure S1 and Figure S2, respectively. ^{1}H NMR (400 MHz, CD_3OD): δ 8.48 (s, 1H), 8.15 (s, 1H), 7.72-7.74 (d, 3H), 7.49 (s, 1H), 7.19-7.21 (d, 1H), 6.33 (s, 1H), 4.49-4.52 (m, 1H), 2.20 (m, 1H), 1.96-1.99 (m, 1H), 1.71 (s, 9H), 1.44 (s, 3H), 1.19(m, 6H). ^{13}C NMR (400 MHz, CD_3OD): δ 174.8, 166.3, 158.6, 148.4, 147.2, 145.8, 142.7, 132.4, 132.0, 130.6, 125.9, 125.3, 117.3, 107.3, 106.8, 81.1, 55.7, 42.0, 33.2, 30.9, 29.1, 26.2, 23.9, 23.8, 21.9, 14.6. HR-ESI-MS, calcd for $C_{27}H_{31}N_4O_4$ $[M]^+$: *m/z* 475.2340; found: *m/z* 475.2328.

A solution of trifluoroacetic acid (2.5 mL) in CH_2Cl_2 (2.5 mL) was added dropwise to the intermediate in 5 mL of CH_2Cl_2 at 0 °C, and then the reaction mixture was stirred at

room temperature for 3 h. The solvent was removed by evaporation under reduced pressure, and the crude product was purified by flash silica gel chromatography eluted with CH_2Cl_2/methanol (v/v, 5/1), affording probe **1** as a crimson solid (30 mg, yield 76%). The ^{1}H NMR and ^{13}C NMR spectra of probe **1** are shown below in Figure S3 and Figure S4, respectively. ^{1}H NMR (400 MHz, CD_3OD): δ 8.79 (d, 1H), 8.36-8.38 (d, 1H), 8.03 (s, 1H), 7.82-7.88 (m, 3H), 7.52-7.54 (d, 1H), 6.69 (s, 1H), 1.77-1.84 (m, 2H), 1.65-1.72 (m, 2H), 1.53-1.60 (m, 2H), 0.98-1.02 (m, 7H). ^{13}C NMR (400 MHz, CD_3OD): δ 176.2, 133.6, 132.9, 132.5, 130.7, 126.5, 125.6, 71.6, 66.8, 55.3, 45.0, 31.9, 30.9, 30.3, 26.0, 23.7, 22.4, 20.4, 14.6, 14.2. HR-ESI-MS, calcd for $C_{22}H_{23}N_4O_2$ $[M]^+$: *m/z* 375.1813; found: *m/z* 375.1815.

General procedure for LAP detection. Unless otherwise stated, all the fluorescence measurements were made according to the following procedure. In a test tube, 10 μL of stock solution of probe **1** and appropriate volume of PBS were mixed, followed by addition of an appropriate volume of LAP or other substance solutions. The mixed solution was adjusted to 1 mL with PBS. After incubation at 37 °C for 20 min, the reaction solution was transferred to a quartz cell of 1-cm optical length to measure fluorescence with $\lambda_{ex/em}$ = 585/625 nm (both excitation and emission slit widths were set to 10 nm). For absorbance measurements, 2 mL of reaction solution was prepared and used. At the same time, a blank solution without LAP was prepared and measured under the same conditions for comparison. Data are expressed as mean ± standard deviation (SD) of three separate measurements.

Detection of LAP in human liver microsomes. In a test tube, 500 μL of human liver microsomes (1 mg/mL) and appropriate volume of PBS were mixed, followed by addition of an appropriate volume of ethanol. The mixed solution was adjusted to 2 mL with PBS, and the final concentration of human liver microsomes was 0.25 mg/mL. After incubation at 37 °C for 1 h, 20 μL of stock solution of probe **1** was added and the mixed solution was incubated 37 °C for additional 25 min. Finally, 2 mL of the reaction solution was transferred to a quartz cell of 1-cm optical length to measure fluorescence with λ_{ex} = 585 nm (both excitation and emission slit widths were set to 10 nm). Data are expressed as mean ± standard deviation (SD) of three separate measurements.

Cell imaging. Cells (HepG2 or A549) were treated at 37 °C for different periods of time (0 – 24 h) with various concentrations of cisplatin (0 – 2 mg/L) in Petri dishes. Before cell imaging, the culture media were removed, and the cells were washed using

DMEM for three times. Then, the cells were incubated with the probe (10 μM) at 37 °C for 20 min in DMEM, washed with DMEM to remove the free probe, and subjected to fluorescence imaging experiments. Unless otherwise noted, data are expressed as mean ± standard deviation (SD) of three separate measurements.

LAP assay by ELISA kit. The microsomes (0.25 mg/mL) were used directly. The cell lysate was prepared according to the following procedure. In a test tube, 1.2×10^5 cells (HepG2 or A549) in 1 mL DMEM was centrifugated at 3000 rpm for 5 min, and then the supernatant was discarded, followed by washing the cells with PBS for three times. After discarding the PBS, 200 μL RIPA Lysis Buffer was added to the test tube, and the tube was left at 0 °C for 20 min. Finally, the tube was centrifugated at 3000 rpm for 10 min, and the supernatant was collected for use. The concentration of LAP in microsomes or cells was determined by measuring the absorbance at 450 nm using a commercial LAP ELISA kit. Briefly, to each well, 40 μL samples or 50 μL standards, 10 μL biotin tagged LAP antibody, and 50 μL enzyme marked reagents were mixed. After incubating the reaction mixture at 37 °C for 60 min, the wells were washed five times with wash solution, and then 50 μL chromogen reagents A and B were added in the wells in succession, followed by incubating for 10 min at 37 °C. Finally, 50 μL stop buffer was added to the wells, and the absorbance of each well was recorded at 450 nm on a Microtiter Plate assay system. The standard curve for LAP detection was constructed following the direction of the kit in the concentration range from 0 to 100 ng/mL, and the corresponding concentrations were calculated according to the standard curve. Data are expressed as mean ± standard deviation (SD) of five separate measurements.

Cytotoxicity assay. Unless otherwise noted, the cytotoxicity of probe **1** or cisplatin was evaluated by the standard MTT assay. Briefly, cells (HepG2 or A549) were seeded in 96-well U-bottom plates at a density of 7000 cells/well, and incubated with probe **1** or cisplatin at varied concentrations at 37 °C for 24 h (72 h for IC_{50} determination). Then, the culture media were discarded, and 0.1 mL of MTT solution (0.5 mg/mL in DMEM) was added to each well, followed by incubation at 37 °C for 4 h. The supernatant was abandoned, and 0.11 mL of DMSO was added to each well to dissolve the formed formazan. After shaking the plates for 10 min, absorbance values of the wells were read with a Microtiter plate assay system at 490 nm. The cell viability rate (V_R) was calculated according to the equation: $V_R = A/A_0 \times 100$ %, where A is the absorbance of the experimental group (the cells treated by probe **1** or cisplatin) and A_0 is the absorbance of

the control group (the cells untreated by probe **1** or cisplatin). Data are expressed as mean ± standard deviation (SD) of five separate measurements.

Transfection. The LAP siRNA was purchased from KeyGEN BioTECH Co. LTD, and the siRNA-transfected HepG2 cells were obtained according to the literature (Kondo *et al. Int. J. Cancer,* **2006**, *118*, 1390).

Western blot. The HepG2 and siRNA-transfected HepG2 cells were lysed with RIPA buffer, and the cell lysates were diluted with PBS to obtain a solution of about 0.5 mg/mL of total proteins. At the same time, the protein standard solution of 0.5 mg/mL was prepared. The protein concentrations and Western blot analysis were performed according to the literatures (Kondo *et al. Int. J. Cancer,* **2006**, *118*, 1390; Fang *et al. J. Am. Chem. Soc.* **2014**, *136*, 226; Li *et al. J. Exp. Bot.* **2011**, *62*, 4763).

Statistical tests. The t values were calculated from formula 1 below, where X_1 and X_2 were the averages of the two samples, n_1 and n_2 were sample capacities, and S_1 and S_2 were standard deviations of the two samples, respectively. The two-sided Student's t-test was used to evaluate the significant difference between the data.

$$t = \frac{X_1 - X_2}{\sqrt{\frac{(n_1 - 1)S_1^2 + (n_2 - 1)S_2^2}{n_1 + n_2 - 2}\left(\frac{n_1 + n_2}{n_1 n_2}\right)}}$$

formula 1

2. Supporting figures

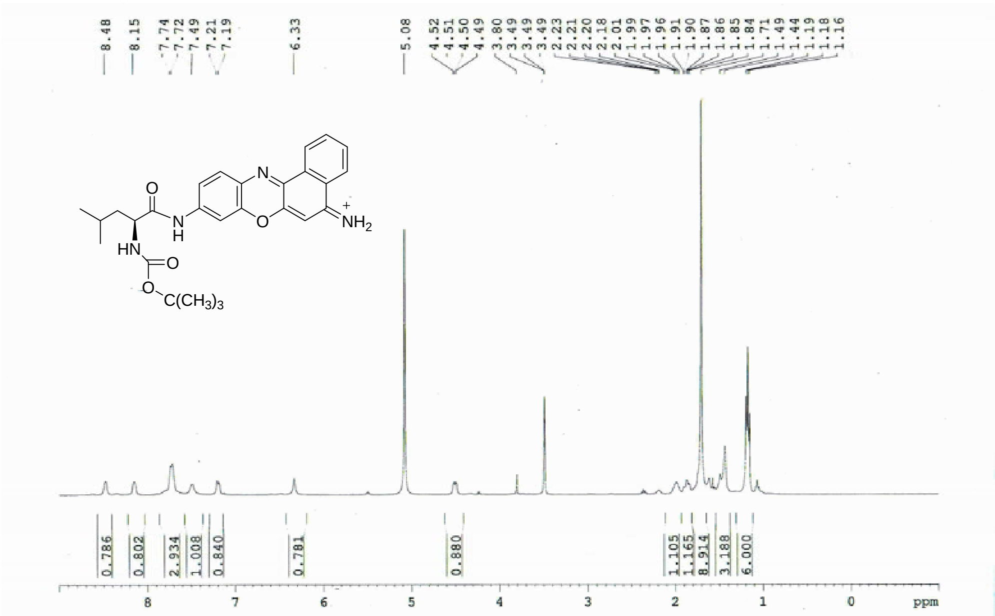

Figure S1. The ^{1}H NMR spectrum of the intermediate in CD_3OD (400 MHz, 298 K).

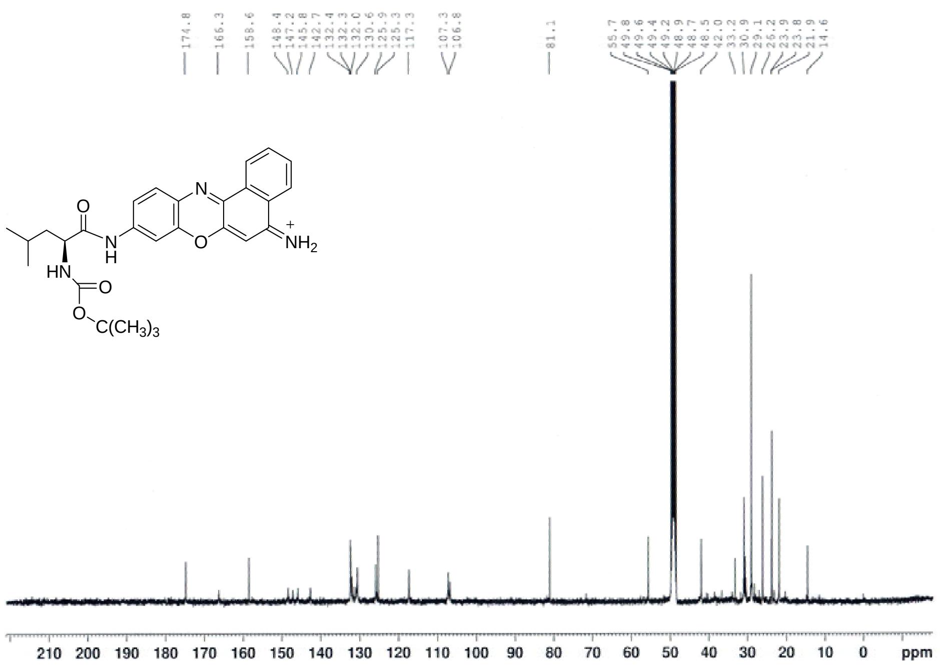

Figure S2. The ^{13}C NMR spectrum of the intermediate in CD_3OD (400 MHz, 298 K).

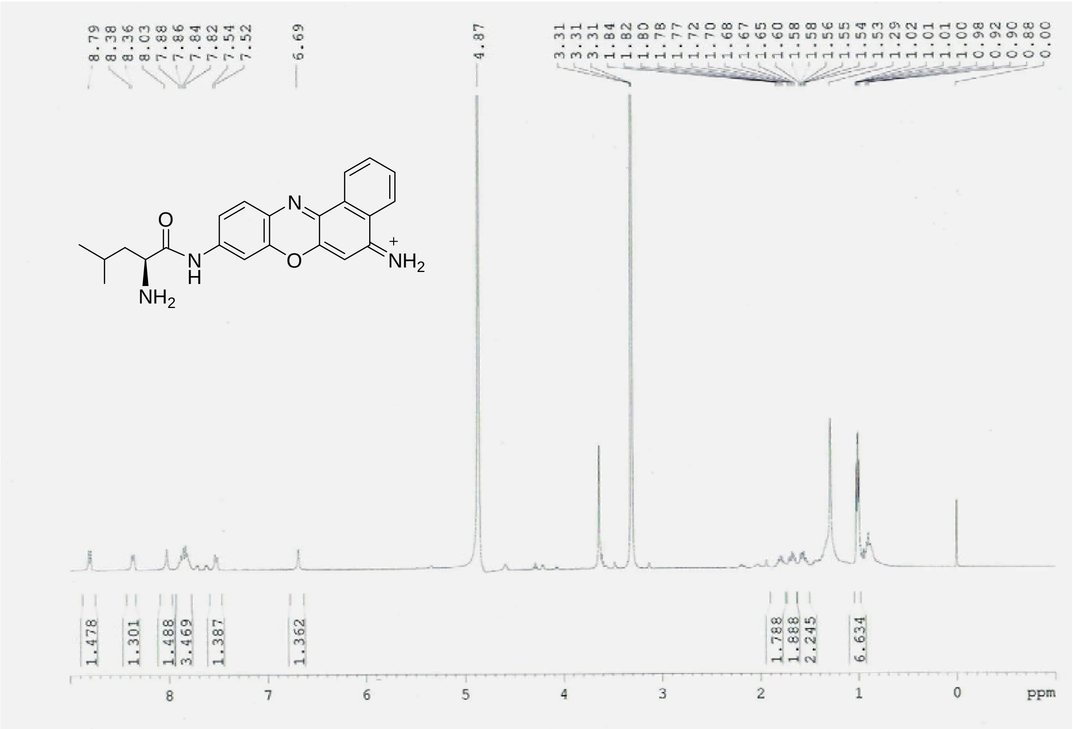

Figure S3. The ^{1}H NMR spectrum of probe **1** in CD_3OD (400 MHz, 298 K).

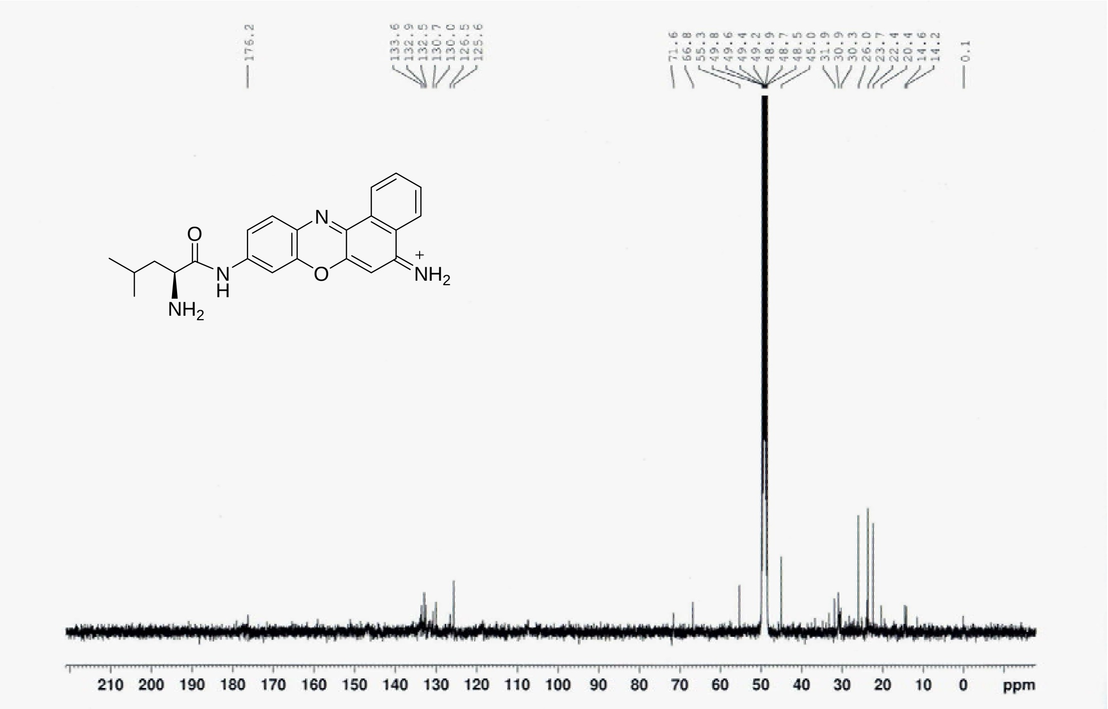

Figure S4. The ^{13}C NMR spectrum of probe **1** in CD_3OD (400 MHz, 298 K).

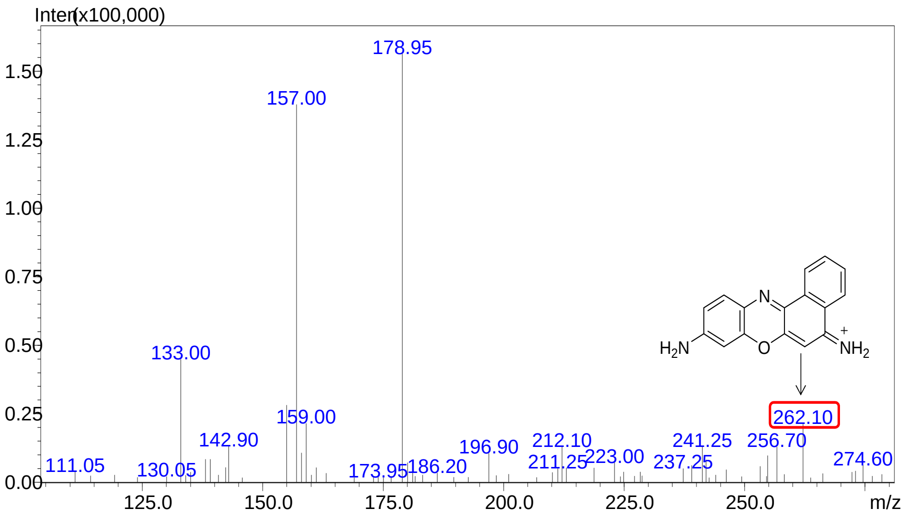

Figure S5. ESI mass spectrum of the reaction products of **1** with LAP.

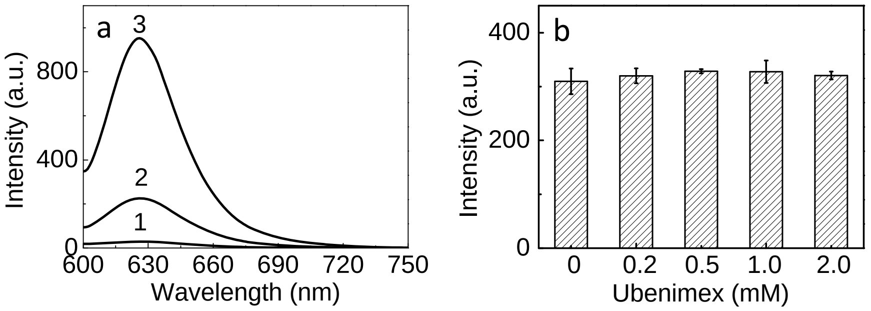

Figure S6. (a) Fluorescence emission spectra (λ_{ex} = 585 nm) of different reaction systems.

1) probe **1** (10 μM) only in PBS; 2) probe **1** (10 μM) + 26 ng/mL LAP + 1.0 mM ubenimex; 3) probe **1** (10 μM) + 26 ng/mL LAP. (b) The effects of ubenimex at varied concentrations on the fluorescence intensity of cresyl violet (5 μM). The reaction was performed at 37 °C in 6.7 mM PBS (pH 7.4). $\lambda_{ex/em}$ = 585/625 nm.

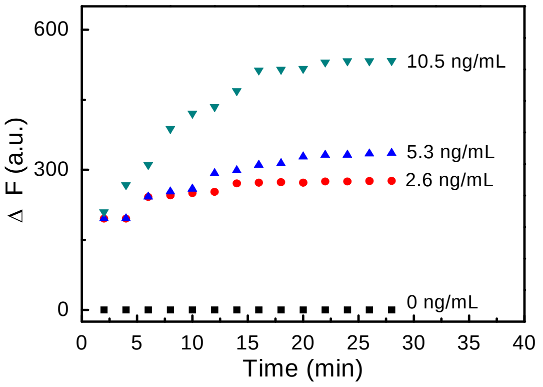

Figure S7. A plot of fluorescence enhancements of probe **1** (10 μM) vs. the reaction time in the presence of varied concentrations of LAP. The reaction was performed at 37 °C in 6.7 mM PBS (pH 7.4). λ_{ex} = 585 nm. As is seen, a reaction time of 20 min may be chosen.

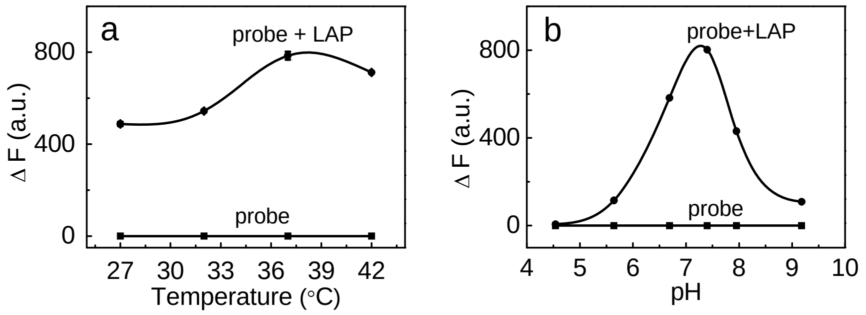

Figure S8. Effects of reaction temperature (a) and pH (b) on the fluorescence of probe **1** (10 μM) in the absence and presence of LAP (26 ng/mL). Conditions: (a) the reaction was performed in PBS (pH 7.4) for 20 min at different temperatures; (b) the reaction was performed at 37 °C for 20 min in PBS with different pH values adjusted with dilute HCl or NaOH. $\lambda_{ex/em}$ = 585/625 nm. From this figure it can be seen that LAP functions well at physiological conditions (pH 7.4 and 37 °C).

Table S1. Comparison of **1** with other small molecular fluorescent probes for LAP

Probe	Detection limit	Wavelength (nm)	Literature
Aminopyridine derivatives	0.05-10 μg/mL	$\lambda_{ex/em}$ = 304-382/396-450	Huang et al, *Anal. Biochem.*, 2009, **391**, 11
Hydroxymethyl rhodamine derivatives	About 30 ng/mL	$\lambda_{ex/em}$ = 495-532/524-573	Sakabe et al, *J. Am. Chem. Soc.*, 2013, **135**, 409
Rhodamine derivatives	No mentioned	$\lambda_{ex/em}$ = 485/535	Grant et al, *J. Biomol. Scre.*, 2002, **7**, 531
2-Dicyanomethylene-3 -cyano-2,5-dihydro-furan derivative	No mentioned	$\lambda_{ex/em}$ = 525/605	Yoon et al, *Bio. Med. Chem. Lett.*, 2011, **21**, 2403
Si-substituted rhodamine derivative	No mentioned	$\lambda_{ex/em}$ = 593/613	Kushida et al, *Bio. Med. Chem. Lett.,* 2012, **22**, 3908
7-L-Leucyl-4-methyl-coumarinylamide	> 1 μg/mL	$\lambda_{ex/em}$ = 330/430	Saifuku et al, *Clin. Chim. Acta*, 1978, **84**, 85
Nile blue derivative	No mentioned	$\lambda_{ex/em}$ = 610/670	Ho et al, *Tetrahedron*, 2006, **62**, 578
Quinazolinone derivatives	No mentioned	$\lambda_{ex/em}$ = 365/495	Zhang et al, *Chem. Eur. J.*, 2010, **16**, 792
Cresyl violet derivative	0.42 ng/mL	$\lambda_{ex/em}$ = 585/625	This work

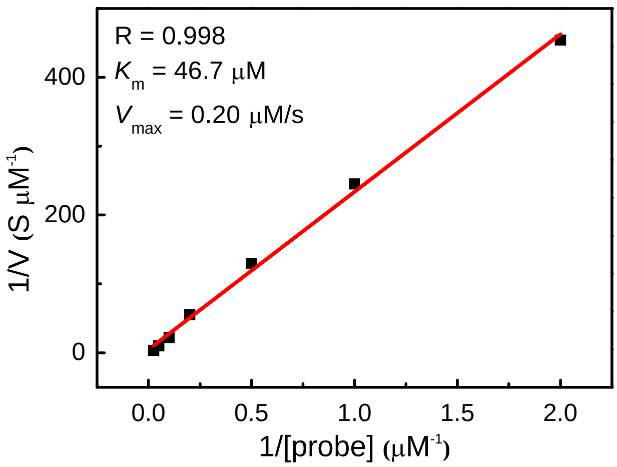

Figure S9. Lineweaver-Burk plot for the enzyme-catalyzed reaction. The Michaelis-Menten equation was described as: $V = V_{max}[\text{probe}]/(K_m+[\text{probe}])$, where V is the reaction rate, [probe] is the probe concentration, and K_m is the Michaelis constant. Conditions: 11 ng/mL LAP, 0.5-40 μM probe **1**, pH 7.4 PBS, temperature 37 °C. $\lambda_{ex/em}$ = 585/625 nm.

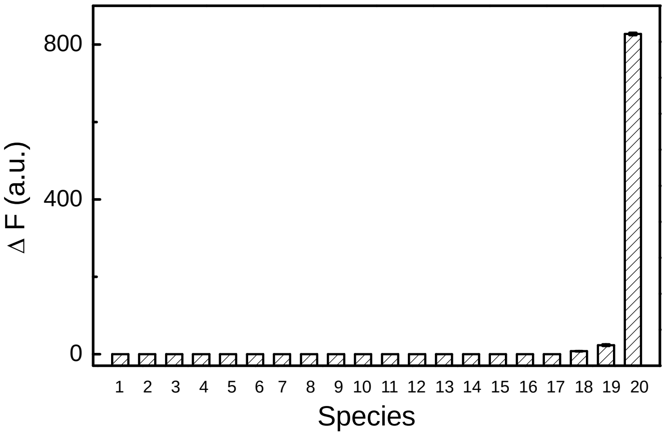

Figure S10. Fluorescence change of probe **1** (10 μM) in the presence of various species: (1) probe only; (2) 150 mM KCl; (3) 2.5 mM $CaCl_2$; (4) 100 μM $ZnCl_2$; (5) 2.5 mM $MgCl_2$; (6) 10 mM glucose; (7) 1 mM vitamin C; (8) 100 μM human serum albumin; (9) 100 μM $\cdot O_2^-$; (10) 100 μM ·OH; (11) 100 μM ClO^-; (12) 1 mM glutamic acid; (13) 1 mM arginine; (14) 5 mM glutathione; (15) 1 mM cysteine; (16) 1 mM homocysteine; (17) 200 μM NADH; (18) 50 U/L glutamic acid dehydrogenase; (19) 50 U/L esterase; (20) 26 ng/mL LAP. All reactions were performed in PBS (pH 7.4) at 37 °C for 20 min. $\lambda_{ex/em}$ = 585/625 nm.

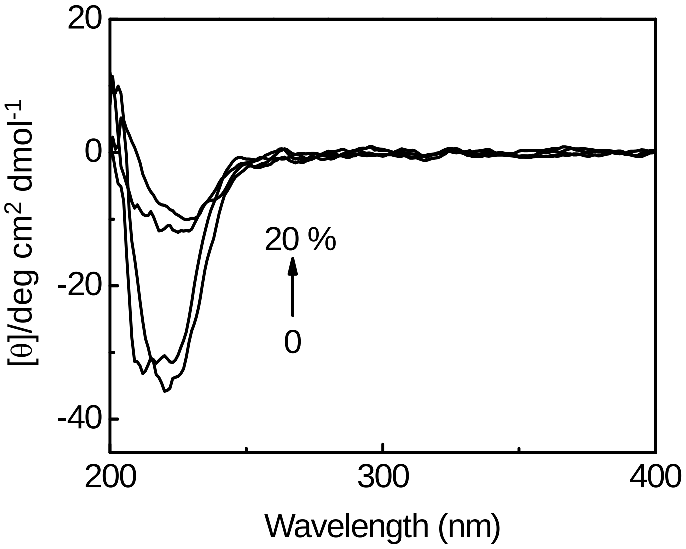

Figure S11. Circular dichroism (CD) spectra of LAP (25 mg/L) at varied concentrations of ethanol [from bottom to top: 0, 10, 15 and 20%(v/v), respectively].

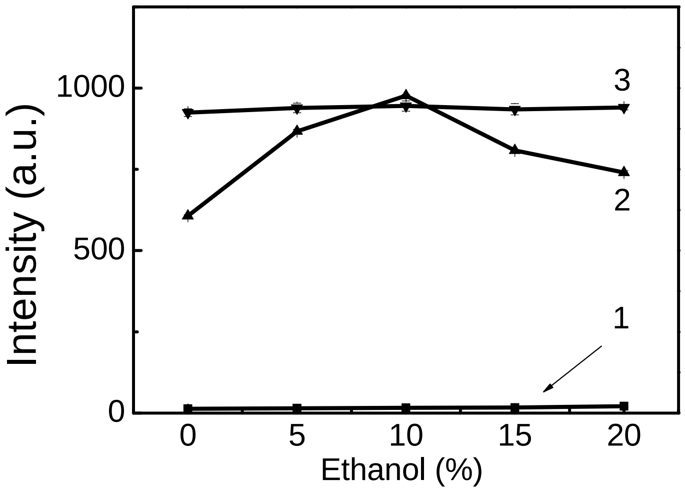

Figure S12. The effects of ethanol concentrations on the fluorescence intensity of different systems. (1): 10 μM probe **1**; (2): 10 μM probe **1** + 0.25 mg/mL microsomes; (3): 10 μM cresyl violet. All the reactions were performed in PBS (pH 7.4) at 37 °C for 20 min. $\lambda_{ex/em}$ = 585/625 nm.

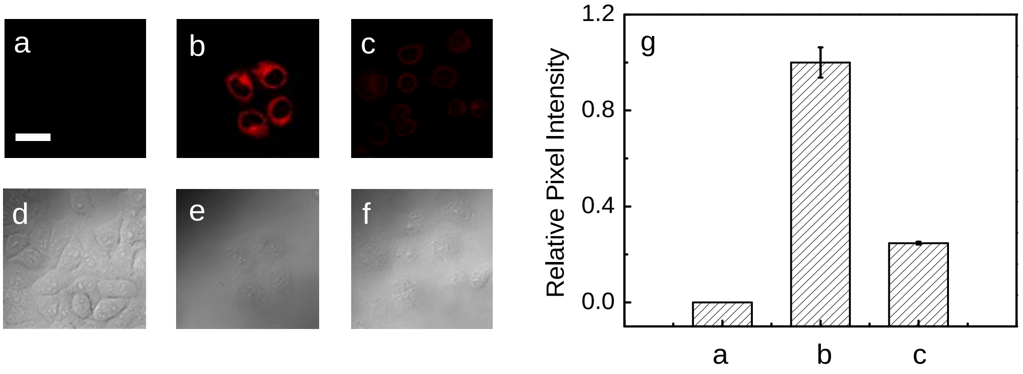

Figure S13. Fluorescent images of LAP in HepG2 cells. (a) HepG2 cells only (control). (b) HepG2 cells treated with the probe (10 μM). (c) HepG2 cells pretreated with 1.0 mM ubenimex and then treated with the probe (10 μM). (d) – (f) Differential interference contrast (DIC) images of the above corresponding samples. (g) Relative pixel intensity measurements (n = 3) from images (a) – (c) by the software ImageJ. The pixel intensity from image (b) is defined as 1.0. Scale bar 20 μm.

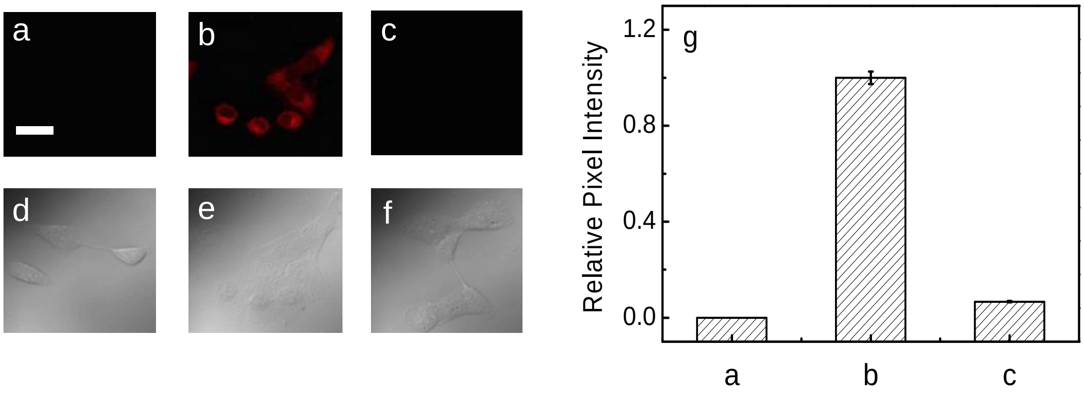

Figure S14. Fluorescent images of LAP in A549 cells. (a) A549 cells only (control). (b) A549 cells treated with **1** (10 μM). (c) A549 cells pretreated with 1.0 mM ubenimex and then treated with **1** (10 μM). (d) – (f) The DIC images of the above corresponding samples. (g) Relative pixel intensity measurements (n = 3) from images (a) – (c) by the software ImageJ. The pixel intensity from image (b) is defined as 1.0. Scale bar, 20 μm.

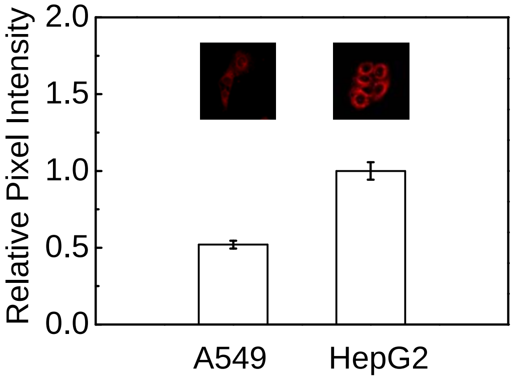

Figure S15. The relative pixel intensity of HepG2 and A549 cells under the same fluorescence imaging conditions.

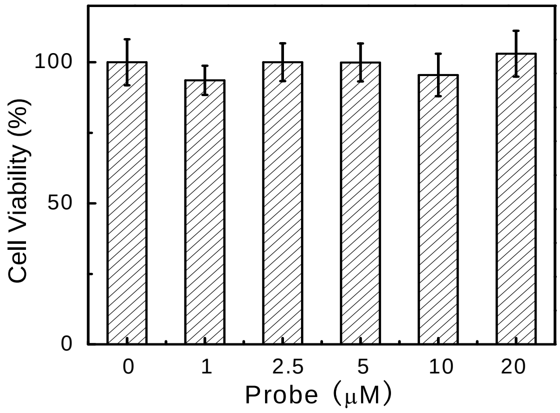

Figure S16. Effects of probe **1** at varied concentrations on the viability of HepG2 cells.

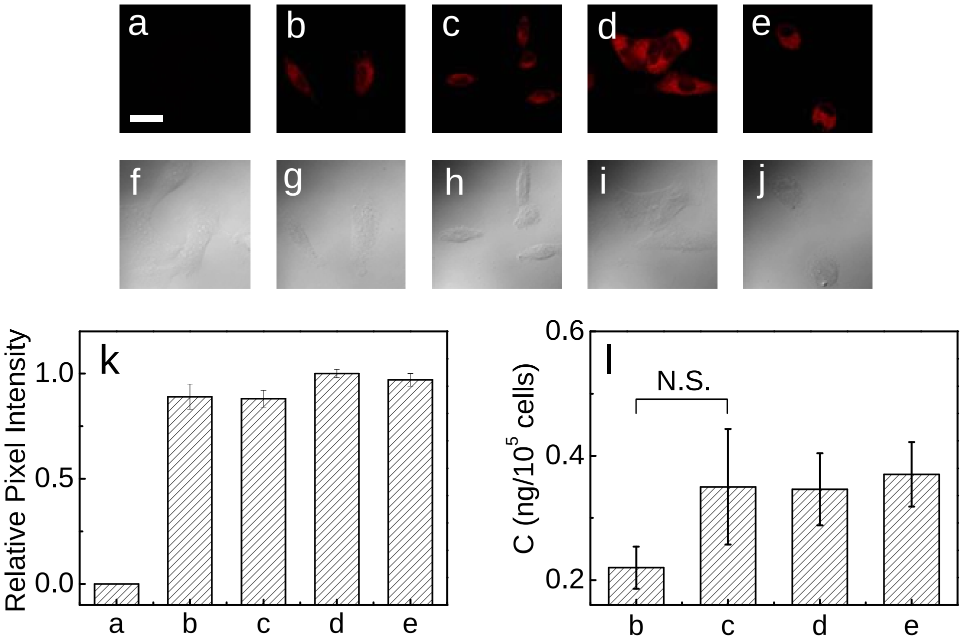

Figure S17. Fluorescence images of LAP in A549 cells. (a) A549 cells only. A549 cells were pretreated with cisplatin at varied concentrations (0, 0.5, 1.0, 2.0 mg/L for images b–e, respectively) for 12 h, and then incubated with **1** (10 μM) for 20 min. Scale bar 20 μm. (f) – (j) The DIC images of the above corresponding A549 cells. (k) Relative pixel intensity measurements (n = 3) from images a–e by using software ImageJ (the pixel intensity from image d is defined as 1.0). (l) The concentrations of LAP in the above A549 cells determined by ELISA kit (N.S.: no significance, p>0.01; two-sided Student's *t*-test).

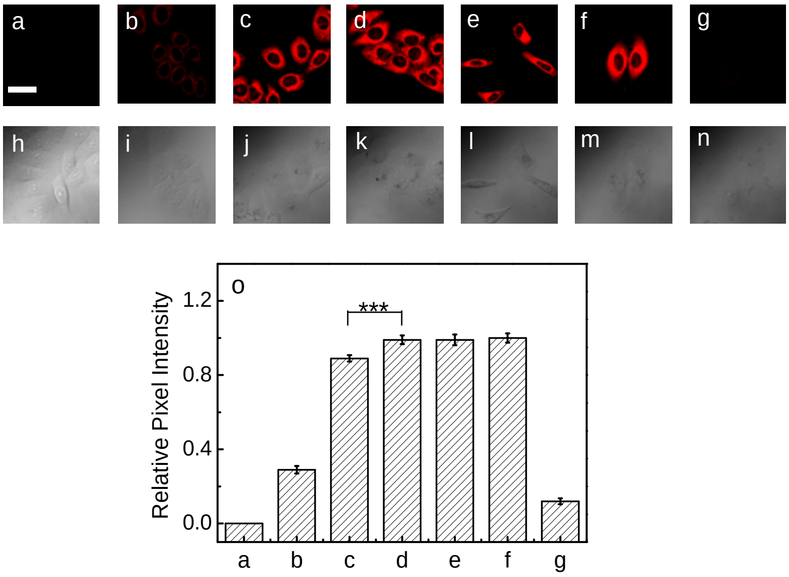

Figure S18. Fluorescence images of HepG2 cells. (a) HepG2 cells only. (b) – (f) The HepG2 cells were pretreated with 1.0 mg/L cisplatin for 0, 2, 6, 12 and 24 h, respectively,

and then incubated with the probe (10 μM) for 20 min. (g) HepG2 cells pretreated with 1.0 mg/L cisplatin for 12 h, then treated with 1.0 mM ubenimex for 1 h, and finally incubated with the probe (10 μM) for 20 min. (h) – (n) The DIC images of the above corresponding HepG2 cells. (o) Relative pixel intensity measurements (n = 3) from images a – g by using the software ImageJ (the pixel intensity from image d is defined as 1.0; *** p<0.001, two-sided Student's *t*-test). Scale bar 20 μm.

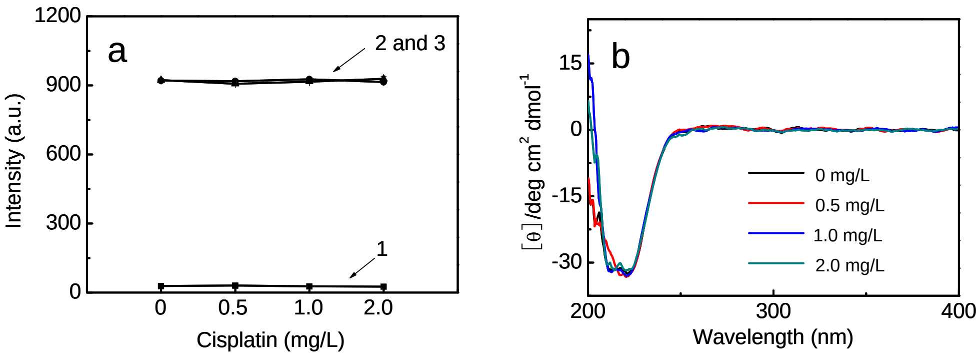

Figure S19. (a) Effects of cisplatin on the fluorescence of (1) probe **1** (10 μM), (2) cresyl violet (10 μM), and (3) the reaction system (10 μM probe **1** + 26 ng/mL LAP). All the reactions were performed in PBS (pH 7.4) at 37 °C for 20 min. $\lambda_{ex/em}$ = 585/625 nm. (b) CD spectra of LAP (25 mg/L) at varied concentrations of cisplatin (0 - 2.0 mg/L). As is seen, these CD spectra are nearly identical, indicating that cisplatin scarcely affects the structure of LAP.

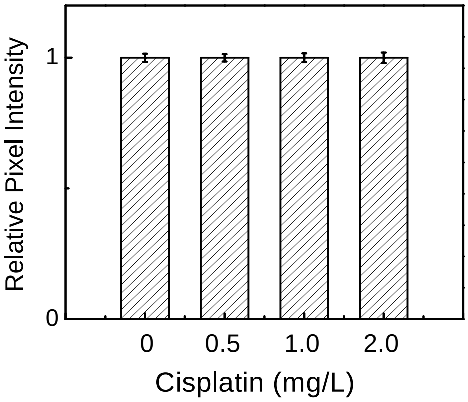

Figure S20. Effects of cisplatin at varied concentrations on the background fluorescence of HepG2 cells (for brevity, the fluorescence images are omitted; the pixel intensity from the control group without cisplatin is defined as 1.0).
